# Public Health and Public Opinion

**Published:** 1918-11-02

**Authors:** 


// The Hospital

The Workers' Newspaper of Administrative Medicine and Institutional Life, Administration, National Insurance and Health.

No. 1691, Vol. LXV. SATURDAY, NOVEMBER 2, 1918. PRICE TWOPENCE

### PUBLIC HEALTH AND PUBLIC OPINION.

The annual report of the Medical Officer to the Local Government Board is, even in ordinary circumstances, a document of great importance, as it necessarily deals with what is of vital concern to the national welfare. In the conditions which exist to-day the importance of such a report has an emphasis that can hardly be overstated. The community generally has been aroused to the intimate relation that exists between personal health and efficiency on the one hand and national capacity on the other. What was formerly regarded as a matter for the individual is seen to be a common interest. From such a conclusion has followed the conviction that to an increasing extent there must be intervention by the State, not only in reference to the promotion of disease by direct measures but also by the encouragement and endowment of enterprises directed to scientific research. Not only must disease, where possible, be prevented, but also health must be promoted. Still more, it is becoming more and more clearly seen that the individual sufferer is within the national claim, and that means for his restoration to the position of a working unit cannot be excluded from the sphere of communal responsibility. It is from these movements of public opinion that there have issued demands for the establishment of a Ministry of Health and for a better organisation and equipment of the Health Services of the country. Such demands are not at all likely to weaken, and it is morally certain that legislative efforts will be made to satisfy them. There lies indeed immediately before us an era in which large changes will be adopted, and these changes will touch both central and local health administrative authorities, voluntary and other hospitals, the medical profession, and various other organisations concerned with the recognition, promotion and cure of disease. It is many years since, at a Lord Mayor's banquet, the Prime Minister of the day recommended as a national motto, *Sanitas, sanitas, omnia sanitas*. There seems just now a fair promise that the advice is to be accepted and to be put into practice.

Existing circumstances therefore combine to heighten the importance of accurate knowledge, for the questions on hand relate to practical concerns, and no solution will be satisfactory which does not recognise the hard facts of experience. Sir Arthur Newsholme's report for 1917-18 is particularly appropriate in this respect. It is characterised by the wide knowledge, reasoned conclusions, and philosophic atmosphere which experience has taught us to expect from the writer, and it ought to be studied by everyone who desires to take a useful part in promoting a more efficient and more penetrating application of the laws of health to the nation generally. The brief summary of what has been accomplished in the last forty years may be more particularly recommended to those who appear to think that hitherto little has been attempted and hardly anything has been done. A second division of the report is engaged with a contemplation of the future, and here, though the writer is necessarily restrained by his official position from discussing controversial issues and legislative proposals, he sounds no uncertain note on the principles which must be accepted if success is to be achieved. A great deal is possible if the country is willing to pay the necessary price, and it is useful to have both these considerations presented clearly and emphatically by one who speaks with special authority, for in the end public opinion will be the dominant factor. The experts must educate their masters, and must educate them not only to the conviction that national health may be improved and that such improvement is highly desirable. Another step is necessary—namely, that the means to secure the improvement are well worth the price they will cost. It is as a good national investment as well as a national opportunity that schemes for the enlargement of preventive and of curative medicine must be presented. The economist equally with the philanthropist must be engaged in the crusade.

That great things have been accomplished in the last forty years is quite beyond question. Typhus fever, which used to be a scourge of crowded communities, has practically disappeared. Enteric fever is moving to the same end. In 1871 it was responsible for over 12,000 deaths, while in 1916, with a population increased by 50 per cent., the deaths numbered only 1,137. The expectation of life at birth, which in 1871-80 was just over forty years, has been increased by fully ten years, and the annual deaths in England and Wales during the period 1910-12 were, on an average, 234,955 fewer than they would have been had the population been subjected to the death-rates of the period 1871-80. For the most part it may be safely said these measures of progress must be placed to the credit of general sanitary administration. The future demands that this administration shall be continued and shall be made more searching. It shows in the forefront the need for greater concentration on the housing problem, where, without doubt, rests the responsibility not only for particular evils, but also for much general physical disability. It presents, too, the need for an attack on specific groups of disease by a combination of scientific agencies and methods. Such common ills as influenza, tuberculosis, pneumonia, measles, and whooping-cough offer themselves as well within the list of preventable diseases, and in these and other directions preventive medicine awaits and expects many triumphs. Sir Arthur Newsholme defines the opportunity and shows how it may be grasped. His report is an educational influence of high value, and it will not lose its reward.

